# The emerging landscape of *in vitro* and *in vivo* epigenetic allelic effects

**DOI:** 10.12688/f1000research.11491.1

**Published:** 2017-12-07

**Authors:** Christopher Gregg

**Affiliations:** 1 University of Utah School of Medicine, Salt Lake City, UT, USA

**Keywords:** non-genetic allelic effects/imprinting/gene regulatory networks/gene expression

## Abstract

Epigenetic mechanisms that cause maternally and paternally inherited alleles to be expressed differently in offspring have the potential to radically change our understanding of the mechanisms that shape disease susceptibility, phenotypic variation, cell fate, and gene expression. However, the nature and prevalence of these effects *in vivo* have been unclear and are debated. Here, I consider major new studies of epigenetic allelic effects in cell lines and primary cells and *in vivo*. The emerging picture is that these effects take on diverse forms, and this review attempts to clarify the nature of the different forms that have been uncovered for genomic imprinting and random monoallelic expression (RME). I also discuss apparent discrepancies between *in vitro *and *in vivo* studies. Importantly, multiple studies suggest that allelic effects are prevalent and can be developmental stage- and cell type-specific. I propose some possible functions and consider roles for allelic effects within the broader context of gene regulatory networks, cellular diversity, and plasticity. Overall, the field is ripe for discovery and is in need of mechanistic and functional studies.

## Understanding gene regulatory networks at the allele level: recipes for cellular, anatomical, physiological, and behavioral phenotypes

Specific gene expression programs in the genome evolved to orchestrate different biological processes, including developmental processes, metabolic processes, and other cellular processes ^1^. The gene regulatory networks that govern gene expression programs are modular and hierarchically organized. They include highly conserved and essential subcircuits, called kernels, as well as various different logic gates and feedback loops to control gene expression in a precise temporal and spatial manner ^1– 3^. The explosion of interest in gene regulation over the past several years has been driven by the recognition that genetic and epigenetic variations in noncoding regulatory elements shape disease risk and phenotypic variation ^4, 5^, and the evolution of new phenotypes frequently involves changes to cis-regulation rather than changes to protein sequence ^6, 7^.

Defining the architecture and logic of the gene regulatory networks and gene expression programs that control different biological processes is challenging. A rare example of a relatively well-defined gene regulatory network in human cells is that controlling embryonic stem cell (ESC) pluripotency ^8^. However, in most cases, our understanding of the gene regulatory networks that control the development and function of the myriad of different cell types in the brain and body has just begun. Enticingly, beyond mechanisms for cell fate, studies of gene regulatory mechanisms in the nervous system have the potential to define gene regulatory networks and gene expression programs that control the development of specific features of behavior, such as particular social behavior traits, anxiety states, and different cognitive and sensorimotor abilities. However, while our understanding of and interest in gene regulatory networks is growing, most approaches assume that the maternal and paternal alleles for a given gene are expressed and regulated equally. Here, I discuss recent and growing evidence for diverse forms of non-genetic effects that cause alleles to be differentially expressed and consider some implications for understanding the regulatory mechanisms and gene expression programs governing cell fate and mammalian phenotypes.

## Genomic imprinting and the differential expression of maternal and paternal alleles at the cellular level

Epigenetic allelic effects that cause maternal and paternal alleles to be expressed differently *in vivo* are best understood from studies of canonical genomic imprinting ^9^, random X-inactivation in females ^10, 11^, allelic exclusion of immunoglobulins ^12^, and RME of clustered protocadherins ^13^ and olfactory receptors ^14^. Many of these cases of established *in vivo* epigenetic allelic effects involve genes with a uniquely clustered organization in the genome. However, others have found evidence for a broader landscape of epigenetic allelic effects in the genome ^15– 17^, although this research area is new, rapidly evolving, and debated. Below, I discuss recent studies that have advanced our understanding of allelic effects and refer readers seeking a more comprehensive literature review to the aforementioned articles.

**Figure 1.**
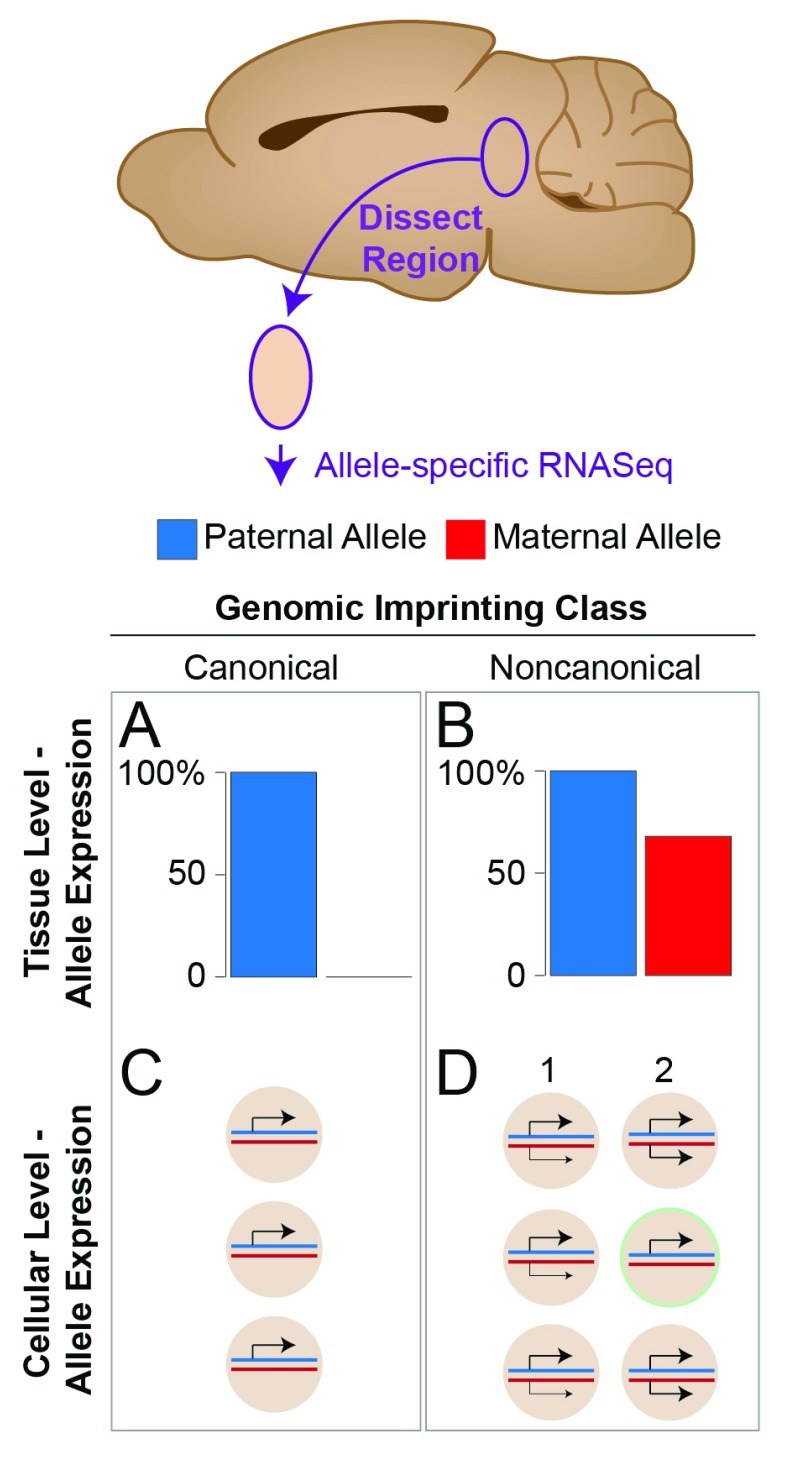
Schematic depiction of canonical versus noncanonical genomic imprinting identified in the mouse. Canonical and noncanonical imprinting was characterized in different mouse tissues by using RNA-Seq in which allele expression was profiled in a piece of tissue dissected from the brain or in another tissue. ( **A**) In this chart, canonical imprinting manifests as complete silencing of one parent’s allele (silent maternal allele shown). ( **B**) In contrast, noncanonical imprinting manifests as a significant bias to express one parental allele at a higher level than the other parental allele (paternal allele bias shown). ( **C**) At the cellular level, canonical imprinting involves complete silencing of one allele in all cells expressing the gene. ( **D**) Noncanonical imprinting may involve either (1) an allelic bias in each cell or (2) allele silencing in a subpopulation of cells in the tissue. Distinguishing between these models (1 versus 2) is an active area of research.

Genomic imprinting is an important phenomenon that causes maternal and paternal alleles to be differentially expressed in offspring. I and others previously described noncanonical imprinting (also referred to as parental allelic biases), which involves maternal or paternal allele expression biases at the tissue level ^17– 20^, in contrast to the allele-silencing effects exhibited by canonical imprinted genes ^18^ ( Figure 1). Some early studies overestimated ^21, 22^ or underestimated ^23, 24^ the prevalence of these effects in the mouse genome. Noncanonical imprinting is less robust and more variable between different individuals than canonical imprinting, and therefore sensitive methods and sufficient statistical power are required to detect these effects accurately ^18– 20, 25^. It is now clear that noncanonical imprinting is a bona fide, highly reproducible epigenetic allelic effect that is especially enriched in the brain and more prevalent than canonical imprinting in the mouse genome ^18, 20^. Furthermore, noncanonical imprinting can shape offspring phenotypes ^17, 18^ and therefore there is a strong motivation to learn more about it.

For many genes, the relative strength of noncanonical imprinting changes between different brain regions and tissue types ^18, 19^. For example, two enzymes that synthesize catecholamines in the brain—tyrosine hydroxylase and dopa decarboxylase ( *Ddc*)—exhibit noncanonical imprinting involving a maternal allele expression bias in the arcuate nucleus (ARN), dorsal raphe nucleus (DRN), and locus coeruleus, but the imprinting effect is not observed in the ventral tegmental area (VTA) for either enzyme ^18^. For *Ddc*, the imprinting is especially strong in the ARN. Overall, these results suggest that noncanonical imprinting is likely influenced by the cellular composition of the target brain region (or tissue) and reflects highly cell type-specific allelic effects in the brain. In support of this interpretation, nascent RNA *in situ* hybridization revealed that brain regions in the mouse that have stronger noncanonical imprinting for *Ddc* are associated with more brain cells that exhibit monoallelic expression, while cells in the VTA, where the imprinting is absent, exhibit biallelic expression ^18^. Thus, an emerging picture is that at least some noncanonical imprinting cases shape maternal and paternal allele expression in a cell type-dependent manner ( Figure 1D).

Other new studies have begun to further clarify the complexities of imprinting at the cellular level in mice. Stelzer and colleagues recently developed a novel reporter of cellular genomic methylation effects that involves placing a differentially methylated region of interest in front of the minimal imprinted promoter region for the gene, *SNRPN*, driving the expression of a green fluorescent protein or tdTomato reporter ^26^. If the differentially methylated region is unmethylated, the reporter is expressed, and if it is methylated, the reporter is silent. With this technology, Stelzer and colleagues recently investigated DNA methylation dynamics at the cellular level *in vivo* for a differentially methylated region that controls imprinting at the *Dlk1-Dio3* imprinted gene cluster ^27^. The study revealed highly cell type- and tissue-specific imprinting as well as imprinting changes during development. In the brain, mosaic methylation of the differentially methylated region was observed in dopaminergic neurons and Purkinje neurons and other cell populations; loss of parent-specific methylation was observed in neural stem cells, consistent with previous work ^28^, and variation between individuals was also found. Thus, the authors discovered that imprinted DNA methylation is more dynamic and varied at the cellular level than was previously known. The field is gaining a deeper appreciation for the complexity of imprinting at the cellular level and, although there is substantial precedence in the literature for such effects ^17^, the prevalence and function of cell type-specific imprinting remain unclear. New *in situ* hybridization-based strategies to resolve allele-specific expression at the cellular level have provided an expanded tool kit for the field to study these effects ^18, 29– 31^. In the brain, cell type-specific maternal and paternal imprinting may shape the development and function of specific brain cells and circuits to modulate particular aspects of offspring brain function and behavior. The identity of the cells, circuits, and brain functions that are impacted and the mechanisms involved are important areas for research.

Currently, less is known about cell type-specific imprinting and noncanonical imprinting in humans. Recent efforts to identify imprinted genes from Genotype-Tissue Expression (GTEx) consortium data were designed to uncover canonical imprinting that involves robust monoallelic expression that is consistent among individuals ^32, 33^. This strategy was necessary because parental genome information was not available to phase the RNA-Seq reads according to parental allele and to avoid various potential artifacts. Nonetheless, these studies have begun to define the landscape of imprinting in the human body and tissue-specific imprinting was found. One study reported relatively more imprinted genes in the human brain compared with other tissues ^32^. As in the mouse, future work analyzing human imprinting at the cellular level is also likely to reveal new information. Additionally, imprinting in the mouse brain is most prevalent and robust in the hypothalamus and in monoaminergic nuclei ^18, 21, 34^, but, other than the hypothalamus, few subcortical regions were included in the GTEx studies, indicating another important area for further study in humans.

## New and diverse forms of epigenetic allelic effects uncovered *in vitro* and *in vivo*

**Figure 2.**
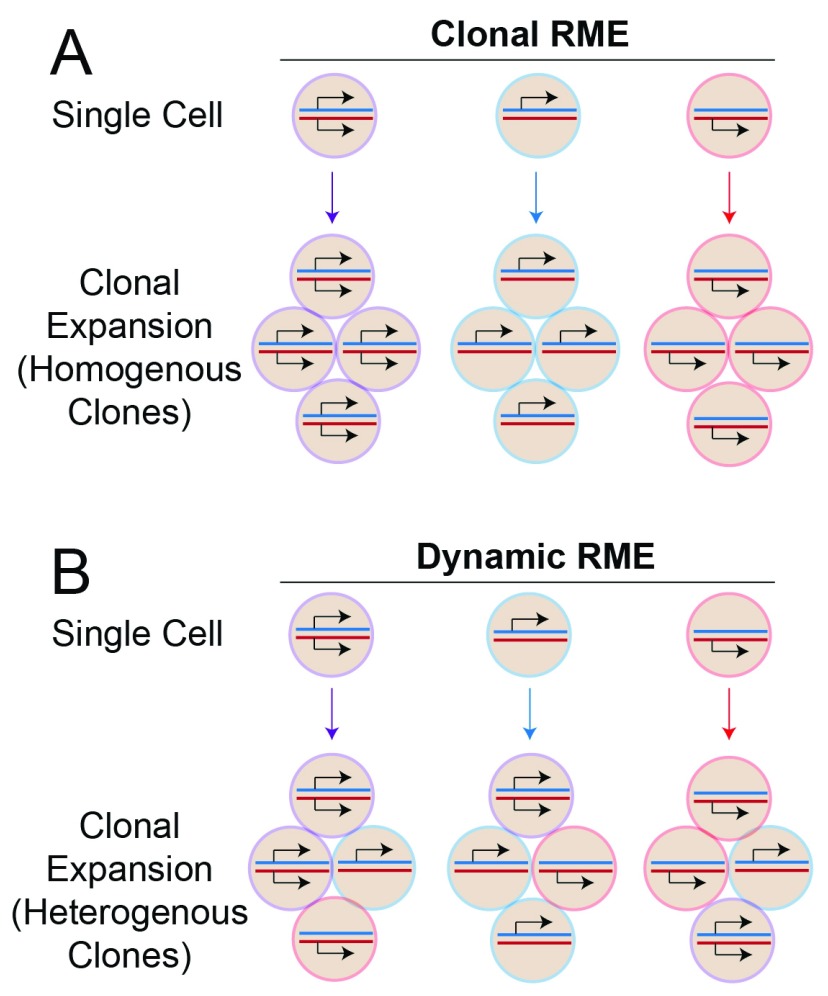
Schematic depiction of clonal versus dynamic random monoallelic expression (RME). ( **A**) Clonal RME is identified when a single cell is expanded to form a colony of daughter cells and each daughter cell has the same allele expression pattern as the original parent cell. Typically, studies of this phenomenon use cell lines and expand them clonally and then profile allelic expression from the entire batch of cells in the clone. They find that some clones exclusively express one allele, others are biallelic, and others express only the other allele (homogenous clones). ( **B**) Dynamic RME occurs when a single cell is expanded to form a colony but the individual cells in the colony have different allelic expression patterns (heterogeneous clones). This phenomenon is detectable only by using single-cell transcriptome analysis and cannot be identified from profiles of the whole batch of cells in the clone, as was done in previous cell line studies reporting widespread clonal RME.

Beyond imprinting, evidence exists for other forms of epigenetic allelic effects, although the nature and prevalence of these effects *in vivo* are debated ^15, 16^ and some new studies have improved our understanding. Widespread RME on the autosomes was first described in human lymphoblastoid cell lines by Gimelbrant and colleagues ^35^. The initial description of this phenomenon indicated similarities to random X-inactivation, such that the monoallelic effect was inherited by daughter cells derived from a single precursor and therefore is clonal. It was estimated that clonal RME impacts 5–15% of genes in human and mouse lymphoblastoid cell lines ( Figure 2A) ^35, 36^. Furthermore, a chromatin signature involving H3K36me3 (activating) and H3K27me3 (repressive) marks was found to distinguish RME genes from other autosomal genes in lymphoblastoid cell lines and then was applied to identify RME genes in human cells and tissues. These studies led the authors to estimate that 20–30% of human genes are subject to RME ^37, 38^. Interestingly, genes with this chromatin signature are more genetically variable in humans ^39^ and appear to be resistant to pathogenic variants impacting expression levels ^40^. This body of work suggests that clonal RME shapes the expression of a large but defined subset of autosomal genes with implications for understanding human genetic variation. However, some other studies suggest a different picture (see below).

Two studies of RME using a similar strategy in mouse ESC lines uncovered a related but more dynamic picture ^41, 42^. In these studies, RME impacted relatively few genes in ESCs but became more prevalent following differentiation into neural progenitor cells, ultimately impacting hundreds of genes. Once established, the monoallelism for a given gene is stable in the neural progenitor cell lines, indicating clonal RME. Thus, the findings from these two major studies suggest that RME is not fixed for specific genes but can change developmentally.

While studies of RME in cell lines yielded a provocative new picture of widespread clonal RME on the autosomes, others have challenged these conclusions and the prevalence of clonal RMEs ^16^. Furthermore, large consortiums studying human allele-specific expression effects *in vivo* concluded that most allele expression differences are explained by genetic variants (expression quantitative trait loci) rather than epigenetic effects ^43, 44^. Similarly, while allelic differences in DNA methylation and chromatin composition are widespread *in vivo* ^45, 46^ and *in vitro* ^5, 47^, these effects also have frequently been attributed to genetic variation ^48– 50^. Thus, *in vitro* versus *in vivo* studies yielded an apparent discrepancy regarding the nature and prevalence of epigenetic allelic effects. While a number of possible explanations exist, recent studies have added some new information.

A new study of RME using single-cell transcriptome profiling in primary cells has challenged previous findings in cell lines regarding the prevalence of clonal RME for mouse and human autosomal genes ^51^. Earlier studies using single-cell transcriptome profiling found RME effects, but few effects were inherited by daughter cells ^16^. A point of note is that over 80% of stochastic allelic expression in single-cell RNA-Seq is potentially due to technical noise ^52^. Thus, this approach is arguably best suited for ruling out clonal RME rather than discovering new RME effects in single cells. Nonetheless, the approach is suitable for the main conclusions drawn in this new study ^51^. Rather than analyzing cell lines, Reinius and colleagues isolated primary mouse fibroblasts and human T cells and expanded single cells to form clones *in vitro* ^51^. By performing single-cell RNA-Seq profiling for individual cells within a clone, they were able to show that clonal RME is very rare, impacting less than 1% of genes in mouse fibroblasts or human T cells, and is associated with genes expressed at very low levels. The identity of the genes impacted in different clones frequently differed. On the other hand, RME that differs between individual cells within a clone, referred to as dynamic RME ( Figure 2B), is frequent and impacts about 13% of genes in fibroblasts and 60–85% of genes in human T cells. The results of this study indicate that clonal RME is very rare *in vivo*, in agreement with the authors’ previous assessment of the field ^16^, but reveals that dynamic RME is frequent and more prevalent in human T cells than mouse fibroblasts. The authors also provide evidence that dynamic RME is related to the transcriptional activity within a cell.

**Figure 3.**
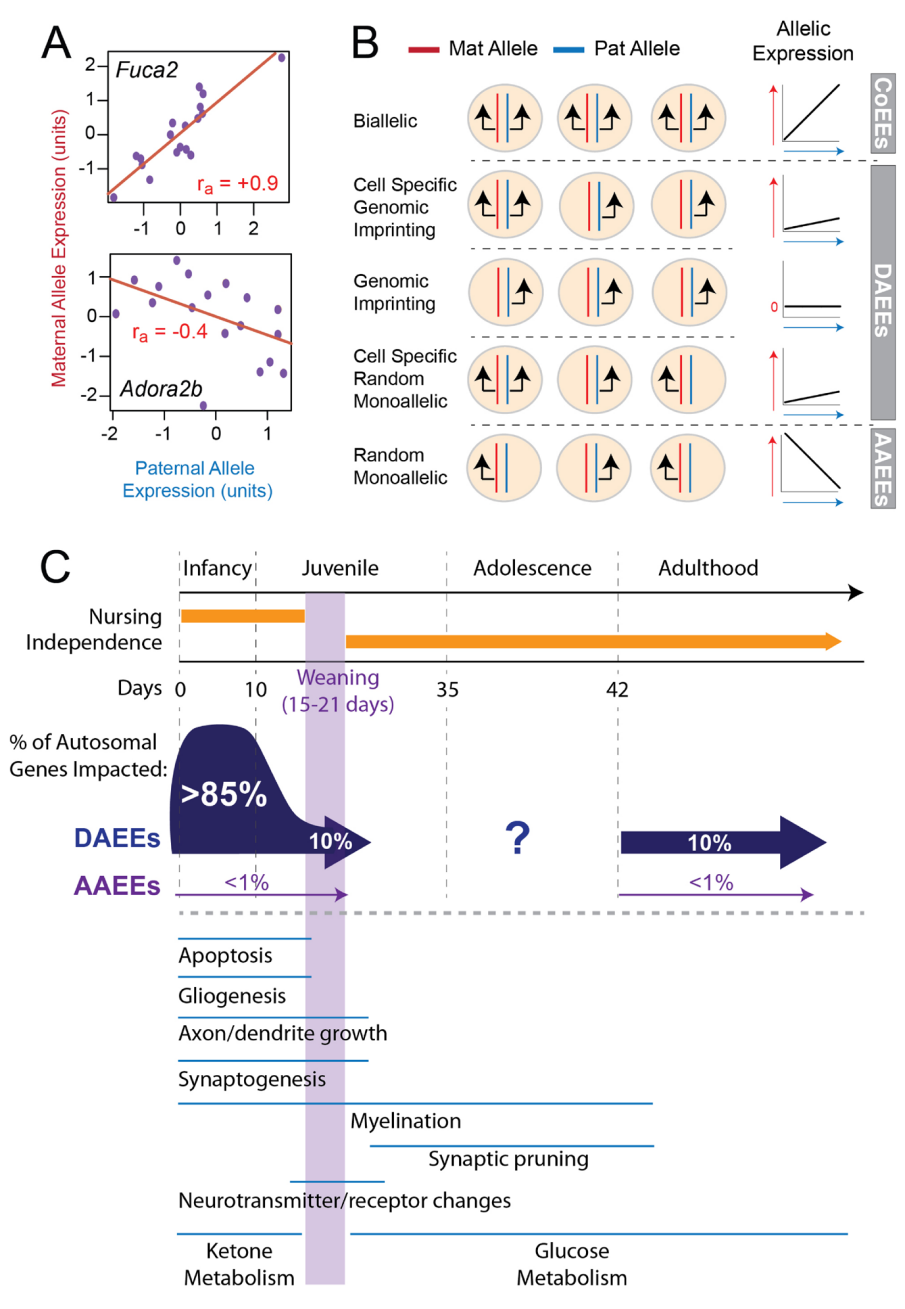
RNA-Seq approach to screen for high-confidence, non-genetic differential allele expression effects *in vivo*. ( **A**) The approach involves RNA-Seq profiling of maternal and paternal allele expression levels across a population of individuals and examining the correlated expression of the two alleles across the population. For example, *Fuca2* exhibits highly correlated allelic expression, while the maternal and paternal alleles for *Adora2b* are negatively correlated. Statistical modeling is performed to estimate the effects of technical noise, biological variation, and genetic variation on the data. The resulting statistic identifies high-confidence, non-genetic allelic effects in a genome-wide manner for any tissue. ( **B**) This *in vivo* screening approach can detect diverse forms of allelic effects. Biallelic expression at the cellular level is expected to manifest as highly correlated allelic expression. Clonal random monoallelic expression (RME) that is similar to X-inactivation will manifest as a negative allele correlation (antagonistic allele expression effects, or AAEEs), since more maternal allele-expressing cells arise at the expense of paternal allele-expressing cells and vice versa. Genome imprinting and cell-specific imprinting or RME will manifest as a weak correlation or no correlation between the alleles; we refer to these cases more generally as differential allele expression effects (DAEEs). ( **C**) Profiling of non-genetic allelic effects in the postnatal day 5 (P5) and P15 and adult mouse DRN revealed major developmental differences. Most genes exhibit evidence for high-confidence DAEEs in the P5 DRN, but these effects are reduced by P15 and in adults such that only 10% of autosomal genes exhibit DAEEs at these older ages. AAEEs are rare *in vivo* and impact less than 1% of all autosomal genes expressed. The *in vivo* developmental shift in non-genetic allelic effects is presented relative to other major developmental milestones and processes in the mouse brain. We applied a similar approach to study DAEEs in the primate brain. CoEE, co-expression effect.

In a recent study, my colleagues and I also sought to gain a deeper understanding of the different forms of non-genetic allelic effects that exist *in vivo* ^53^. We devised a robust genomics and statistical methodology that tests the null hypothesis that the maternal and paternal alleles for a given gene are equally co-expressed (or correlated) across different RNA-Seq biological replicates from a particular brain region or tissue type ( Figure 3A). This screening approach has the potential to uncover a wide range of different allelic effects *in vivo*, including imprinting, clonal RME, dynamic RME, and other possible effects ( Figure 3B). Genes with clonal RME *in vivo* will typically have negatively correlated allelic expression ( Figure 3B), and, as expected, many randomly inactivated X-linked genes in females are detected with this signature. Genes with biallelic expression have positively correlated allele expression, and, finally, genes with canonical imprinting, cell-specific imprinting, cell-specific RME, or other possible allelic effects will manifest with low or no allele correlation, which we refer to broadly as differential allele expression effects ( Figure 3B). The screen was performed in the DRN in postnatal day 5 (P5) and P15 and adult female mice and in the ARN in the hypothalamus, the liver, and skeletal muscle as well as in the juvenile DRN of female cynomolgus macaques.

Our study revealed that over 85% of genes exhibit high-confidence differential allele expression in the developing P5 DRN, indicating profound differences in the expression patterns of maternal and paternal alleles at this stage, and very few cases involved imprinting ( Figure 3C). In P15 juveniles and adults, only about 10% of genes are impacted and most genes shift toward allele co-expression at these later developmental stages ( Figure 3C). We further show that genes with allele co-expression predominantly exhibit biallelic expression at the cellular level but that genes with differential allele expression predominantly exhibit monoallelic expression. These results reveal a developmental shift in allelic effects in mice, which is associated with the progression of neuronal and glial cell differentiation, cell and synaptic pruning, and circuit formation and maturation in the brain ( Figure 3C). Together with previous *in vitro* studies of RME ^41, 42^, an emerging picture is that non-genetic allelic effects are relatively infrequent in ESCs, increase in frequency in neural progenitors, are highly prevalent in the neonatal brain, and decrease in the mature brain ^41, 42, 53^. These observations suggest that many allelic effects are more than just stochastic transcriptional noise in genes expressed at a low level. In fact, genes with *in vivo* differential allelic expression are not expressed at lower levels than genes without these effects ^53^ and they appear to be a major feature of developmental gene expression programs.

While we found that differential allelic expression is frequent *in vivo*, we also found that very few autosomal genes exhibit evidence for clonal RME effects that are similar to random X-inactivation, which manifests as a negative allele correlation with our approach. Many X-linked genes in females exhibit a negative allele correlation, but fewer than 15 autosomal genes exhibit evidence for these types of effects in the mouse in any of the tissues examined ^53^. These findings may be consistent with the results of Reinius and colleagues ^16, 51^, since they indicate that strict clonal RME is indeed rare *in vivo*. Some of the differential allelic expression effects we observe *in vivo* could involve clonal RME in a subpopulation of cells *in vivo*, as was observed in cell lines ^35, 36, 41, 42^, or dynamic RME ^16, 51^; we currently do not know the underlying mechanisms involved. Importantly, dynamic RME simply refers to allelic effects that differ between cells in the same clone ^51^ and the temporal stability of these effects is not known. Overall, further studies are needed to investigate clonal versus dynamic RME *in vivo* and in specific cell lineages and the temporal stability of these effects for different genes.

## Potential functional roles for allelic effects in shaping cellular diversity and gene regulatory network plasticity

**Figure 4.**
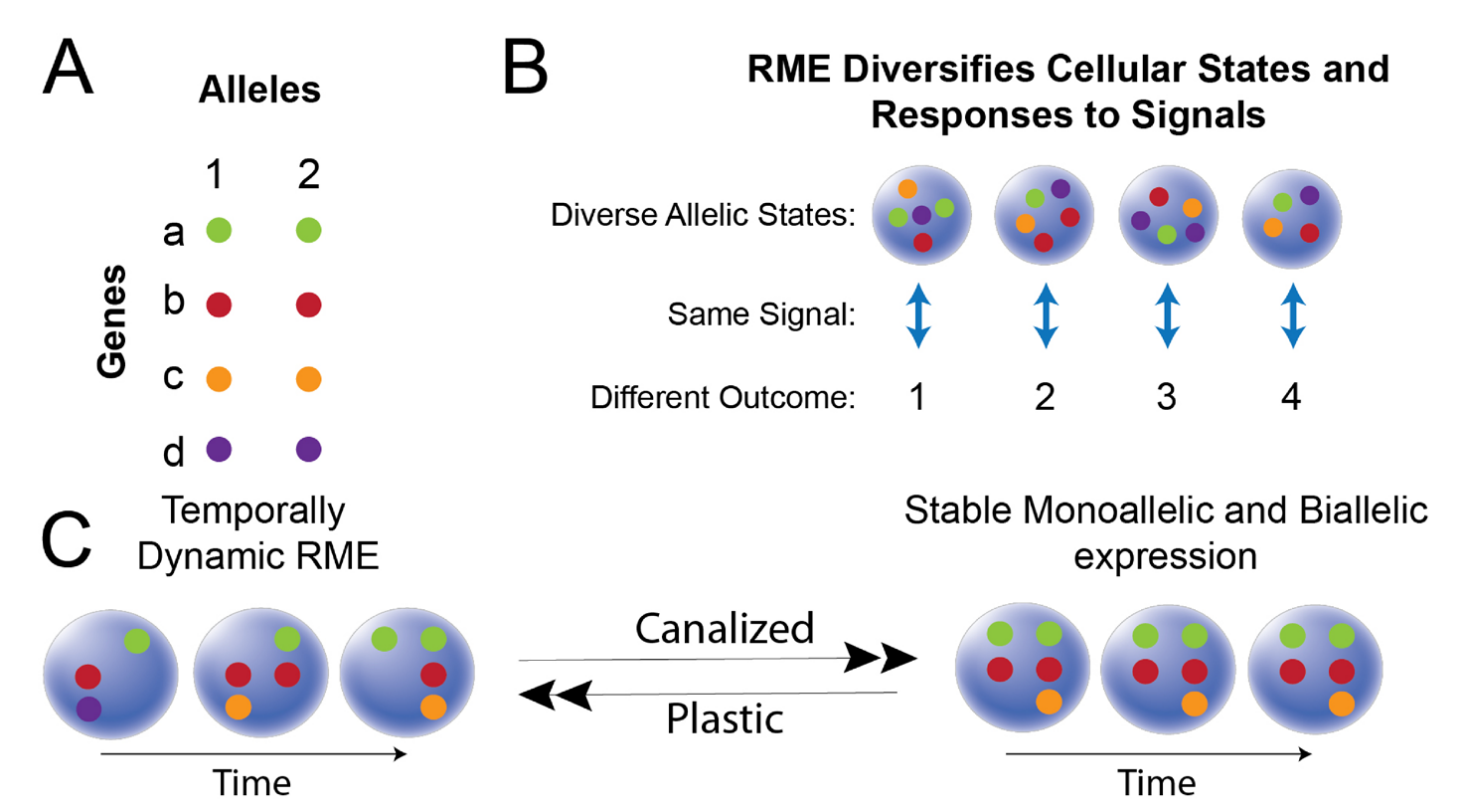
Possible functions for different forms of random monoallelic expression (RME) in promoting cellular diversity and plasticity in mammalian cells. ( **A**) Schematic of two alleles (1 and 2) for four genes (a–d). ( **B**) A population of four cells is diversified by clonal or dynamic RME such that each cell expresses a different allelic combination. Exposure of the population to a signal in the environment is predicted to result in different outcomes for each cell (1, 2, 3, 4). Some cells will be in a state that responds better (more quickly or correctly) to the signal than other cells. ( **C**) Cellular and gene transitions from a temporally dynamic RME state to a stable biallelic or monoallelic (clonal RME or imprinting) state are predicted to shape plasticity versus canalization for gene networks within a cell. The temporally dynamic RME state for different genes is a predicted state of increased plasticity because the cell has access to different allelic combinations that are maintained in a poised state, but these combinations are no longer available once a gene and cell commit to a stable allelic expression state.

Studies of stochastic gene expression effects across otherwise identical prokaryotic and eukaryotic cell populations have shown that cellular gene expression variability can function to diversify an otherwise homogeneous cell population ^54– 57^. Others have argued that stochastic epigenetic variation in humans and mice promotes phenotypic variation ^58– 60^, providing a powerful evolutionary strategy to cope with changing and unpredictable environments by diversifying a population of organisms. Similarly, clonal and dynamic RME may be an important source of variation, placing otherwise similar cells into different states and thereby introducing diversity and plasticity into the population ( Figure 4A and B). By increasing the diversity of gene expression programs in a cell population, some cells may respond differently to the same signal, but at least some will respond appropriately ( Figure 4B). Thus, the population is prepared for cues arising in a changing and unpredictable cellular environment.

At the level of individual cells, commitment to stable biallelic or monoallelic expression states (imprinting or clonal RME) is expected to promote the canalization of gene regulatory networks, thereby committing cells to a particular fate ( Figure 4C). In contrast, if dynamic RME effects can change temporally for different genes in the same cell, such that different allelic combinations are maintained in a poised state and available to be expressed, this mechanism could function to increase plasticity and expand the landscape of gene regulatory networks and programs available to the cell ( Figure 4C). Interestingly, transitions between different cellular states during development have recently been shown to involve a destabilization of gene expression, such that the cell can respond to diverse environmental cues during the transition from one state into a new state ^61^. I speculate that the presence versus absence of dynamic RME could reflect this type of destabilization, promoting cellular plasticity and permitting the formation of new epigenetic states within a cell ^62^. Seemingly consistent with this prediction is the finding that about 60–85% of genes in human T cells exhibit dynamic RME ^51^, since T cells are a highly plastic cell type that must respond to unpredictable environmental cues ^63^. In contrast, only 13% of genes exhibit dynamic RME in mouse fibroblasts, which presumably are less plastic than T cells ^51^. Interestingly, several examples of RME have been described in immune cells over the years ^15^; however, the function of effects other than allelic exclusion is currently unknown.

Enticingly, a model for allelic effects in regulating gene expression plasticity and diversity predicts that allelic effects could have roles in shaping cell fate decisions in the developing brain and how environmental factors, such as stress, diet, drugs, infection, and disease, impact cells in the brain and body. However, in apparent opposition to this model is the observation that ESCs have a relatively low frequency of RME ^41, 42^ yet they are a highly plastic cell type. It is possible that the pluripotent state does not benefit from allelic diversity in the same way as more committed cell types. Alternatively, allelic effects may serve other functions that remain to be uncovered and are not related to promoting gene regulatory network plasticity and cellular diversity.

## Interactions between allelic effects and genetic variation at the cellular level

**Figure 5.**
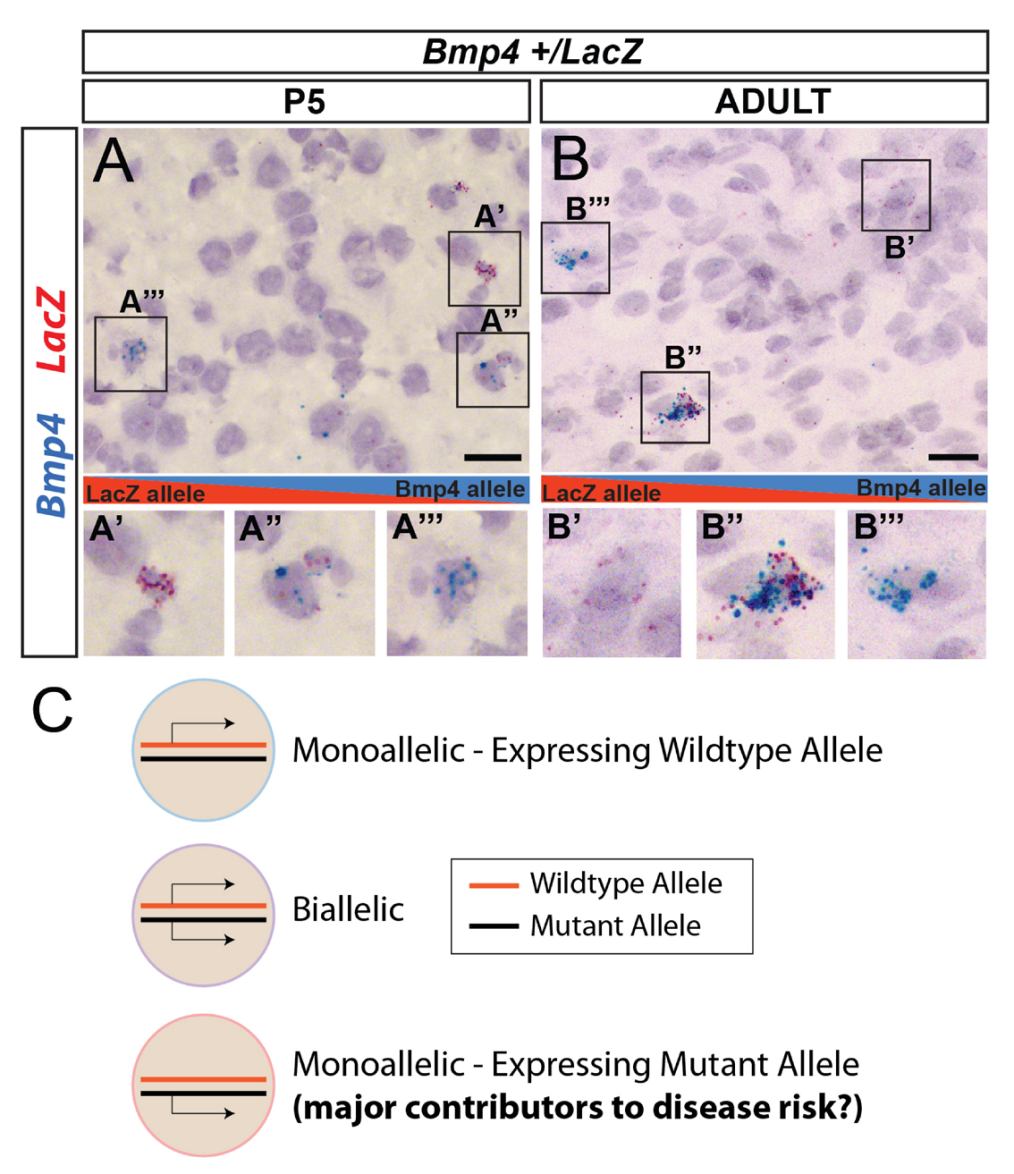
Interactions between non-genetic allelic effects and heterozygous variants can shape genetic architecture at the cellular level. ( **A**, **B**) Single-molecule mRNA *in situ* hybridization for the mutant ( *LacZ*, red) and wild-type ( *Bmp4*, blue) alleles in a heterozygous knockout reporter *Bmp4 ^LacZ/+^* mouse line. Images of the postnatal day 5 (P5) and adult mouse brain are shown and reveal a mosaic of cells that preferentially express the mutant allele (red; **A**’ and **B**’), wild-type allele (blue; **A**’’ and **B**’’), and biallelic (red and blue co-expressed; **A**’’’ and **B**’’’) cells. ( **C**) Monoallelic mutant allele-expressing cells might be more dysfunctional than biallelic or monoallelic wild-type allele-expressing cells for some mutations.

When considered in the context of genetic variation, cells exhibiting RME for a gene are diversified by the potential to express not only different combinations of alleles but also different combinations of heterozygous variants. The effect of such genetic allelic diversity across a population of cells is predicted to further contribute to diversity in cellular responses to the environment and physiological state of the organism. We recently demonstrated that a heterozygous mutation in a gene with differential allelic expression, such as *Bmp4*, results in mosaics of cells in the mouse brain, such that some brain cells express the mutated allele, some express the wild-type allele, and some express both alleles ( Figure 5) ^53^. This mosaic pattern was found to differ according to cell type for some genes. However, while our study showed how these effects can interact with genetic variants, we focused on the RNA level and it is unclear whether such effects also manifest at the protein level, which is important to determine in order to fully understand the impact on genetic architecture.

Interactions between epigenetic allelic effects and heterozygous genetic variation could improve our understanding of the factors that drive phenotypic variation in different disorders, such as mental illnesses ^15, 16, 64, 65^. Indeed, we found that differential allelic expression effects exist *in vivo* in the primate brain and impact genes linked to mental illness in the macaque and human brain, including autism-linked genes and huntingtin ^53^. RME for disease-linked genes has also been observed in cell lines ^35, 41, 42, 66^ and recently for the autism-linked gene *FOXP2* in humans ^67^. Allelic effects in the autistic brain have also been identified, although it is unclear whether the effects are genetic or epigenetic in origin ^68^. As others have proposed ^15, 64, 65^, we speculate that cells that preferentially express mutated alleles, or particular combinations of mutated alleles, due to epigenetic allelic effects may play important roles in contributing to disease risk and phenotypic variance ( Figure 5C). However, these ideas remain to be tested.

## Possible links between imprinting and random monoallelic expression

The prevalence of RME *in vivo* provides opportunities for discovery and reflection. In an insightful review by Ohlsson and colleagues in 2001, it was proposed that genomic imprinting and random X-inactivation evolved from stochastic allele expression effects in the genome, perhaps to better coordinate the expression of groups of genes ^69^. With the expanded landscape of RME effects that have been uncovered *in vivo* and *in vitro*, and the characterization of noncanonical imprinting *in vivo*, it is worth revisiting the relationship between RME and imprinting. If some forms of RME function to promote plasticity within cellular gene regulatory networks or cellular diversity or both, then imprinting is expected to constrain these effects, as noted above ( Figure 4C). Thus, noncanonical and canonical imprinting may function to reduce plasticity and cellular diversity, stabilizing particular gene regulatory networks and promoting specific cellular states and phenotypic traits in offspring. The strength of the imprinting effect (for example, canonical versus noncanonical) may be related to the strength of the imprinting constraint placed on the RME effect for some genes. Indeed, preliminary data in our lab suggest that some noncanonical imprinted genes also exhibit RME at the cellular level in the brain (Paul J. Bonthuis and C. Gregg, unpublished observations).

## Uncovering the mechanisms governing non-genetic allelic effects

**Figure 6.**
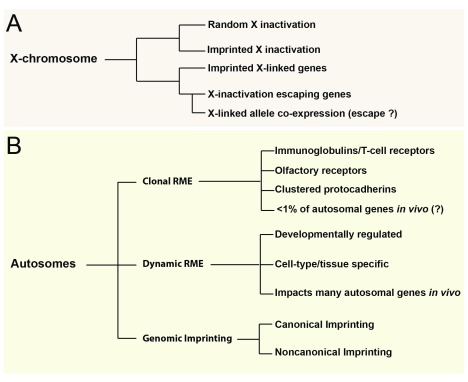
The current landscape of *in vivo* non-genetic allelic effects in mammals ( **A**) Non-genetic allelic effects that impact X-linked genes in females are shown. ( **B**) Effects impacting autosomal genes are shown. Tissue- and age-specific allele co-expression effects for X-linked genes were observed by Huang and Ferris and colleagues ^53^ (2017), but the underlying cause is not yet known. Additionally, the frequency of clonal random monoallelic expression (RME) effects *in vivo* is debated and requires further investigation.

Our review of the literature above indicates that non-genetic allelic effects are prevalent and take on many different forms. We summarize the current *in vivo* landscape of these effects in Figure 6. In most cases, the mechanisms involved are not known. For instance, the mechanisms involved in causing rare clonal RME versus dynamic RME versus biallelic expression are not known. Furthermore, for dynamic RME, we do not know how stable these effects are over time; some may be stable during specific developmental stages and others may be more transient. Our study revealed 335 genes that exhibit differential allelic expression in the mouse DRN across all developmental stages examined ^53^. We also found 69 genes that exhibit differential allelic expression across all tissue types examined (brain, liver, and muscle). However, most *in vivo* effects appear to be developmental stage-, tissue-, and cell type-specific. Chromatin structure and transcription factor dosage are likely to have important roles in regulating these effects. Indeed, in the brain, profound epigenetic changes are known to occur over the course of development and chromatin is highly cell type-specific ^70– 73^.

**Figure 7.**
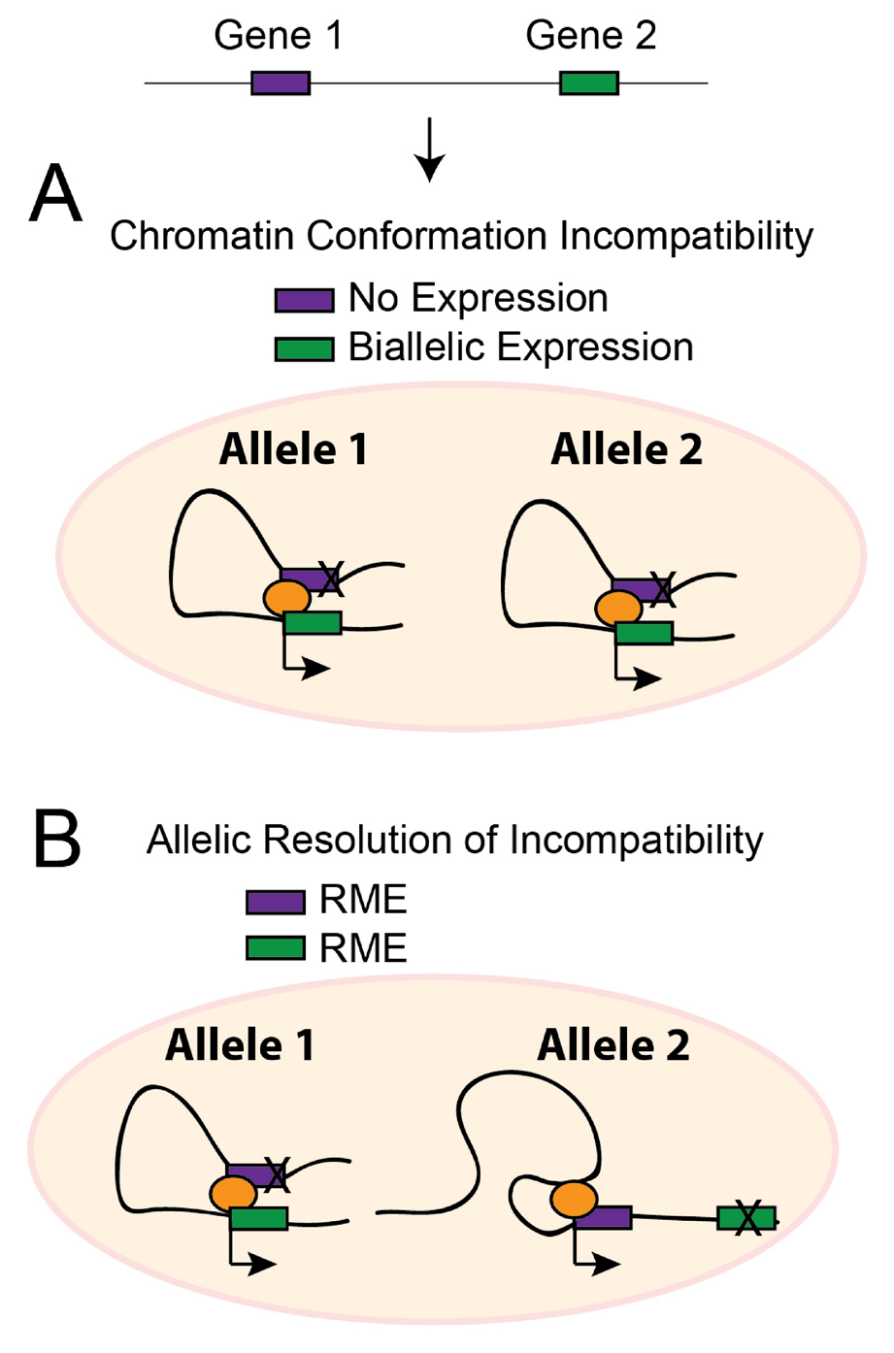
Random monoallelic expression (RME) effects may resolve chromatin conformation incompatibilities in the genome, allowing the activation of diverse gene regulatory networks across a cell population. A schematic representation of a chromatin incompatibility that influences the cellular expression of two genes is shown. Gene 1 and gene 2 compete for a particular chromatin state for their expression. ( **A**) In the absence of RME effects, only one gene can win and is expressed in a biallelic manner in a cell. ( **B**) However, if RME effects are present, then different chromatin structures can form on each allele to resolve the incompatibility and permit the simultaneous expression of both genes in the same cell. This model of RME effects implies that these effects may function to allow cells in a population access to diverse potential gene regulatory networks, thereby further contributing to cellular plasticity.

In one model of RME, chromatin conformation incompatibilities may prevent some genes from being expressed simultaneously from the same allele in the same cell, in which case RME effects would resolve these incompatibilities and permit particular combinations of genes to be expressed simultaneously in the same cell ( Figure 7). Indeed, such allelic incompatibility is known for the imprinted genes *Igf2* and *H19*, which form allele-specific chromatin loops that provide both genes access to the same upstream enhancers ^74, 75^. Allelic competition for enhancers also contributes to singular allelic expression for olfactory receptors in olfactory neurons ^76^ and is a plausible mechanism contributing to other autosomal clonal and dynamic RME effects. RME might also arise due to transcriptional interference if the enhancer or transcriptional start site for one gene is located in a position that disrupts the expression of another gene and the interference is resolved in an allele-specific manner. Indeed, many genes in the genome overlap ^77^, which can result in transcriptional interference leading to allele-specific expression effects, as was shown for some imprinted genes ^78^. Furthermore, about 51% of enhancers reside within the introns or exons of coding genes ^79^ and can regulate the expression of neighboring genes ^80, 81^. Thus, RME might resolve various potential regulatory incompatibilities in the genome, allowing diverse gene regulatory networks to be differentially active or poised in different cells within a cell population.

A new study by Xu and colleagues used ATAC-Seq to uncover the nature of random monoallelic chromatin architecture and DNA accessibility in mouse ESC lines differentiated into neural progenitors ^82^. The results reveal that allele-specific DNA accessibility increases in prevalence following the differentiation of ESCs into neural progenitors and that most allele-specific open chromatin sites occur in promoter regions rather than distal regulatory elements. These effects are stable once they are established at specific genomic sites in clonal neural progenitor cell lines. It may be feasible to adapt this powerful strategy to perform *in vivo* profiling of differential allelic DNA accessibility in tissues and purified cell populations using the statistical methods we recently developed ^53^. The new findings by Xu and colleagues are a major first step toward understanding the mechanisms involved in clonal autosomal RME.

Opportunities for the discovery of novel allelic effects at the chromatin and cellular levels are likely to be plentiful. Early studies revealed that chromosomes occupy specific territories in the nucleus, and elegant studies using fluorescent *in situ* hybridization and chromatin confirmation capture found that genome topology changes in response to cellular differentiation and can vary at the cellular level and between cell types ^83, 84^. At the time, however, little attention appears to have been paid to the relative locations of the maternal and paternal chromosomes in these studies, yet it is clear from the reported data that the two chromosomes frequently occupy different relative positions in the nucleus ^85, 86^. The same appears to be true for the relative positioning of alleles in healthy and diseased cells ^87, 88^. Therefore, at the cellular level, the topological organization, globular structure, and chromatin architecture of maternal and paternal chromosomes may differ frequently, which could reflect differences in gene regulation at the cis or trans level or both. Indeed, recent work in macrophages uncovered dynamic changes from monoallelic to biallelic expression for TNFalpha in response to pro-inflammatory cues, which was associated with changes to the relative location of the alleles in the nucleus ^89^.

## Conclusions

Throughout this article, I have highlighted many exciting opportunities for discovery in this expanding field. I suggest that a deeper understanding of noncanonical imprinting and RME at the cellular level *in vivo* is needed, and I propose that some allelic effects might function to regulate the plasticity versus canalization of gene regulatory networks within a cell and shape cellular diversity within a population. Overall, new studies in the field have uncovered diverse forms of non-genetic allelic effects *in vitro* and *in vivo* and, when heterozygous mutations are present, these effects can shape the expression of mutated versus wild-type alleles at the cellular level, at least at the RNA level. Although the temporal stability of RME has not been established, it appears that many genes have the capacity to move in and out of an RME state. Therefore, unlike imprinting, which impacts a defined subset of genes in the genome, RME appears to be a more general and dynamic property of gene expression, particularly during development. The prevalence of clonal RME *in vivo* appears to be less than was initially thought from cell lines, but more studies are warranted. The prevalence of noncanonical imprinting has now been clarified in mice and shown to impact offspring phenotypes, but the mechanisms and function are undefined and little is known in humans. Overall, many opportunities for new mechanistic and functional investigations exist. The field is poised to improve our understanding of gene regulation and genetic architecture in development, neurobiology, and disease.

## Abbreviations

ARN, arcuate nucleus; Ddc, dopa decarboxylase; DRN, dorsal raphe nucleus; ESC, embryonic stem cell; GTEx, Genotype-Tissue Expression; P, postnatal day; RME, random monoallelic expression; VTA, ventral tegmental area.

## References

[1] Thompson D Regev A Roy S: Comparative analysis of gene regulatory networks: from network reconstruction to evolution. *Annu Rev Cell Dev Biol.* 2015;31:399–428. 10.1146/annurev-cellbio-100913-012908 26355593

[2] Davidson EH: Emerging properties of animal gene regulatory networks. *Nature.* 2010;468(7326):911–20. 10.1038/nature09645 21164479 PMC3967874

[3] Erwin DH Davidson EH: The evolution of hierarchical gene regulatory networks. *Nat Rev Genet.* 2009;10(2):141–8. 10.1038/nrg2499 19139764

[4] Vockley CM Barrera A Reddy TE: Decoding the role of regulatory element polymorphisms in complex disease. *Curr Opin Genet Dev.* 2017;43:38–45. 10.1016/j.gde.2016.10.007 27984826

[5] Roadmap Epigenomics Consortium, Kundaje A Meuleman W: Integrative analysis of 111 reference human epigenomes. *Nature.* 2015;518(7539):317–30. 10.1038/nature14248 25693563 PMC4530010

[6] Wray GA: The evolutionary significance of *cis*-regulatory mutations. *Nat Rev Genet.* 2007;8(3):206–16. 10.1038/nrg2063 17304246

[7] Carroll SB: Evo-devo and an expanding evolutionary synthesis: a genetic theory of morphological evolution. *Cell.* 2008;134(1):25–36. 10.1016/j.cell.2008.06.030 18614008

[8] Boyer LA Lee TI Cole MF: Core transcriptional regulatory circuitry in human embryonic stem cells. *Cell.* 2005;122(6):947–56. 10.1016/j.cell.2005.08.020 16153702 PMC3006442

[9] Bartolomei MS Ferguson-Smith AC: Mammalian genomic imprinting. *Cold Spring Harb Perspect Biol.* 2011;3(7): pii: a002592. 10.1101/cshperspect.a002592 21576252 PMC3119911

[10] Lee JT: Gracefully ageing at 50, X-chromosome inactivation becomes a paradigm for RNA and chromatin control. *Nat Rev Mol Cell Biol.* 2011;12(12):815–26. 10.1038/nrm3231 22108600

[11] Deng X Berletch JB Nguyen DK: X chromosome regulation: diverse patterns in development, tissues and disease. *Nat Rev Genet.* 2014;15(6):367–78. 10.1038/nrg3687 24733023 PMC4117651

[12] Vettermann C Schlissel MS: Allelic exclusion of immunoglobulin genes: models and mechanisms. *Immunol Rev.* 2010;237(1):22–42. 10.1111/j.1600-065X.2010.00935.x 20727027 PMC2928156

[13] Chen WV Maniatis T: Clustered protocadherins. *Development.* 2013;140(16):3297–302. 10.1242/dev.090621 23900538 PMC3737714

[14] Monahan K Lomvardas S: Monoallelic expression of olfactory receptors. *Annu Rev Cell Dev Biol.* 2015;31:721–40. 10.1146/annurev-cellbio-100814-125308 26359778 PMC4882762

[15] Chess A: Monoallelic Gene Expression in Mammals. *Annu Rev Genet.* 2016;50:317–27. 10.1146/annurev-genet-120215-035120 27893959

[16] Reinius B Sandberg R: Random monoallelic expression of autosomal genes: stochastic transcription and allele-level regulation. *Nat Rev Genet.* 2015;16(11):653–64. 10.1038/nrg3888 26442639

[17] Perez JD Rubinstein ND Dulac C: New Perspectives on Genomic Imprinting, an Essential and Multifaceted Mode of Epigenetic Control in the Developing and Adult Brain. *Annu Rev Neurosci.* 2016;39:347–84. 10.1146/annurev-neuro-061010-113708 27145912 PMC5125552

[18] Bonthuis PJ Huang WC Stacher Hörndli CN: Noncanonical Genomic Imprinting Effects in Offspring. *Cell Rep.* 2015;12(6):979–91. 10.1016/j.celrep.2015.07.017 26235621

[19] Perez JD Rubinstein ND Fernandez DE: Quantitative and functional interrogation of parent-of-origin allelic expression biases in the brain. *eLife.* 2015;4:e07860. 10.7554/eLife.07860 26140685 PMC4512258

[20] Crowley JJ Zhabotynsky V Sun W: Analyses of allele-specific gene expression in highly divergent mouse crosses identifies pervasive allelic imbalance. *Nat Genet.* 2015;47(4):353–60. 10.1038/ng.3222 25730764 PMC4380817

[21] Gregg C Zhang J Weissbourd B: High-resolution analysis of parent-of-origin allelic expression in the mouse brain. *Science.* 2010;329(5992):643–8. 10.1126/science.1190830 20616232 PMC3005244

[22] Gregg C Zhang J Butler JE: Sex-specific parent-of-origin allelic expression in the mouse brain. *Science.* 2010;329(5992):682–5. 10.1126/science.1190831 20616234 PMC2997643

[23] Wang X Sun Q McGrath SD: Transcriptome-wide identification of novel imprinted genes in neonatal mouse brain. *PLoS One.* 2008;3(12):e3839. 10.1371/journal.pone.0003839 19052635 PMC2585789

[24] Babak T Deveale B Armour C: Global survey of genomic imprinting by transcriptome sequencing. *Curr Biol.* 2008;18(22):1735–41. 10.1016/j.cub.2008.09.044 19026546

[25] Deveale B van der Kooy D Babak T: Critical evaluation of imprinted gene expression by RNA-Seq: a new perspective. *PLoS Genet.* 2012;8(3):e1002600. 10.1371/journal.pgen.1002600 22479196 PMC3315459

[26] Stelzer Y Shivalila CS Soldner F: Tracing dynamic changes of DNA methylation at single-cell resolution. *Cell.* 2015;163(1):218–29. 10.1016/j.cell.2015.08.046 26406378 PMC4583717

[27] Stelzer Y Wu H Song Y: Parent-of-Origin DNA Methylation Dynamics during Mouse Development. *Cell Rep.* 2016;16(12):3167–80. 10.1016/j.celrep.2016.08.066 27653683 PMC5119552

[28] Ferrón SR Charalambous M Radford E: Postnatal loss of *Dlk1* imprinting in stem cells and niche astrocytes regulates neurogenesis. *Nature.* 2011;475(7356):381–5. 10.1038/nature10229 21776083 PMC3160481

[29] Levesque MJ Ginart P Wei Y: Visualizing SNVs to quantify allele-specific expression in single cells. *Nat Methods.* 2013;10(9):865–7. 10.1038/nmeth.2589 23913259 PMC3771873

[30] Ginart P Kalish JM Jiang CL: Visualizing allele-specific expression in single cells reveals epigenetic mosaicism in an *H19* loss-of-imprinting mutant. *Genes Dev.* 2016;30(5):567–78. 10.1101/gad.275958.115 26944681 PMC4782050

[31] Hansen CH van Oudenaarden A: Allele-specific detection of single mRNA molecules *in situ*. *Nat Methods.* 2013;10(9):869–71. 10.1038/nmeth.2601 23934076 PMC3789122

[32] Babak T Deveale B Tsang EK: Genetic conflict reflected in tissue-specific maps of genomic imprinting in human and mouse. *Nat Genet.* 2015;47(5):544–9. 10.1038/ng.3274 25848752 PMC4414907

[33] Baran Y Subramaniam M Biton A: The landscape of genomic imprinting across diverse adult human tissues. *Genome Res.* 2015;25(7):927–36. 10.1101/gr.192278.115 25953952 PMC4484390

[34] Ivanova E Kelsey G: Imprinted genes and hypothalamic function. *J Mol Endocrinol.* 2011;47(2):R67–74. 10.1530/JME-11-0065 21798993

[35] Gimelbrant A Hutchinson JN Thompson BR: Widespread monoallelic expression on human autosomes. *Science.* 2007;318(5853):1136–40. 10.1126/science.1148910 18006746

[36] Zwemer LM Zak A Thompson BR: Autosomal monoallelic expression in the mouse. *Genome Biol.* 2012;13(2):R10. 10.1186/gb-2012-13-2-r10 22348269 PMC3334567

[37] Nag A Savova V Fung HL: Chromatin signature of widespread monoallelic expression. *eLife.* 2013;2:e01256. 10.7554/eLife.01256 24381246 PMC3873816

[38] Nag A Vigneau S Savova V: Chromatin Signature Identifies Monoallelic Gene Expression Across Mammalian Cell Types. *G3 (Bethesda).* 2015;5(8):1713–20. 10.1534/g3.115.018853 26092837 PMC4528328

[39] Savova V Chun S Sohail M: Genes with monoallelic expression contribute disproportionately to genetic diversity in humans. *Nat Genet.* 2016;48(3):231–7. 10.1038/ng.3493 26808112 PMC4942303

[40] Savova V Vinogradova S Pruss D: Risk alleles of genes with monoallelic expression are enriched in gain-of-function variants and depleted in loss-of-function variants for neurodevelopmental disorders. *Mol Psychiatry.* 2017;22(12):1785–1794. 10.1038/mp.2017.13 28265118 PMC5589474

[41] Gendrel AV Attia M Chen CJ: Developmental dynamics and disease potential of random monoallelic gene expression. *Dev Cell.* 2014;28(4):366–80. 10.1016/j.devcel.2014.01.016 24576422

[42] Eckersley-Maslin MA Thybert D Bergmann JH: Random monoallelic gene expression increases upon embryonic stem cell differentiation. *Dev Cell.* 2014;28(4):351–65. 10.1016/j.devcel.2014.01.017 24576421 PMC3955261

[43] GTEx Consortium: Human genomics. The Genotype-Tissue Expression (GTEx) pilot analysis: multitissue gene regulation in humans. *Science.* 2015;348(6235):648–60. 10.1126/science.1262110 25954001 PMC4547484

[44] Chen L Ge B Casale FP: Genetic Drivers of Epigenetic and Transcriptional Variation in Human Immune Cells. *Cell.* 2016;167(5):1398–1414.e24. 10.1016/j.cell.2016.10.026 27863251 PMC5119954

[45] Schultz MD He Y Whitaker JW: Human body epigenome maps reveal noncanonical DNA methylation variation. *Nature.* 2015;523(7559):212–6. 10.1038/nature14465 26030523 PMC4499021

[46] Leung D Jung I Rajagopal N: Integrative analysis of haplotype-resolved epigenomes across human tissues. *Nature.* 2015;518(7539):350–4. 10.1038/nature14217 25693566 PMC4449149

[47] Dixon JR Jung I Selvaraj S: Chromatin architecture reorganization during stem cell differentiation. *Nature.* 2015;518(7539):331–6. 10.1038/nature14222 25693564 PMC4515363

[48] Kilpinen H Waszak SM Gschwind AR: Coordinated effects of sequence variation on DNA binding, chromatin structure, and transcription. *Science.* 2013;342(6159):744–7. 10.1126/science.1242463 24136355 PMC5502466

[49] Heinz S Romanoski CE Benner C: Effect of natural genetic variation on enhancer selection and function. *Nature.* 2013;503(7477):487–92. 10.1038/nature12615 24121437 PMC3994126

[50] Kasowski M Kyriazopoulou-Panagiotopoulou S Grubert F: Extensive variation in chromatin states across humans. *Science.* 2013;342(6159):750–2. 10.1126/science.1242510 24136358 PMC4075767

[51] Reinius B Mold JE Ramsköld D: Analysis of allelic expression patterns in clonal somatic cells by single-cell RNA-seq. *Nat Genet.* 2016;48(11):1430–5. 10.1038/ng.3678 27668657 PMC5117254

[52] Kim JK Kolodziejczyk AA Ilicic T: Characterizing noise structure in single-cell RNA-seq distinguishes genuine from technical stochastic allelic expression. *Nat Commun.* 2015;6:8687. 10.1038/ncomms9687 26489834 PMC4627577

[53] Huang WC Ferris E Cheng T: Diverse Non-genetic, Allele-Specific Expression Effects Shape Genetic Architecture at the Cellular Level in the Mammalian Brain. *Neuron.* 2017;93(5):1094–1109.e7. 10.1016/j.neuron.2017.01.033 28238550 PMC5774018

[54] Kaern M Elston TC Blake WJ: Stochasticity in gene expression: from theories to phenotypes. *Nat Rev Genet.* 2005;6(6):451–64. 10.1038/nrg1615 15883588

[55] Balázsi G van Oudenaarden A Collins JJ: Cellular decision making and biological noise: from microbes to mammals. *Cell.* 2011;144(6):910–25. 10.1016/j.cell.2011.01.030 21414483 PMC3068611

[56] Raj A van Oudenaarden A: Nature, nurture, or chance: stochastic gene expression and its consequences. *Cell.* 2008;135(2):216–26. 10.1016/j.cell.2008.09.050 18957198 PMC3118044

[57] Raj A Rifkin SA Andersen E: Variability in gene expression underlies incomplete penetrance. *Nature.* 2010;463(7283):913–8. 10.1038/nature08781 20164922 PMC2836165

[58] Feinberg AP Irizarry RA: Evolution in health and medicine Sackler colloquium: Stochastic epigenetic variation as a driving force of development, evolutionary adaptation, and disease. *Proc Natl Acad Sci U S A.* 2010;107 Suppl 1:1757–64. 10.1073/pnas.0906183107 20080672 PMC2868296

[59] Alter MD Hen R: Is there a genomic tone? Implications for understanding development, adaptation and treatment. *Dev Neurosci.* 2009;31(4):351–7. 10.1159/000216546 19546572

[60] Alter MD Rubin DB Ramsey K: Variation in the large-scale organization of gene expression levels in the hippocampus relates to stable epigenetic variability in behavior. *PLoS One.* 2008;3(10):e3344. 10.1371/journal.pone.0003344 18836535 PMC2556388

[61] Mojtahedi M Skupin A Zhou J: Cell Fate Decision as High-Dimensional Critical State Transition. *PLoS Biol.* 2016;14(12):e2000640. 10.1371/journal.pbio.2000640 28027308 PMC5189937

[62] Acar M Becskei A van Oudenaarden A: Enhancement of cellular memory by reducing stochastic transitions. *Nature.* 2005;435(7039):228–32. 10.1038/nature03524 15889097

[63] DuPage M Bluestone JA: Harnessing the plasticity of CD4 ^+^ T cells to treat immune-mediated disease. *Nat Rev Immunol.* 2016;16(3):149–63. 10.1038/nri.2015.18 26875830

[64] Gendrel AV Marion-Poll L Katoh K: Random monoallelic expression of genes on autosomes: Parallels with X-chromosome inactivation. *Semin Cell Dev Biol.* 2016;56:100–10. 10.1016/j.semcdb.2016.04.007 27101886

[65] Eckersley-Maslin MA Spector DL: Random monoallelic expression: regulating gene expression one allele at a time. *Trends Genet.* 2014;30(6):237–44. 10.1016/j.tig.2014.03.003 24780084 PMC4037383

[66] Jeffries AR Collier DA Vassos E: Random or stochastic monoallelic expressed genes are enriched for neurodevelopmental disorder candidate genes. *PLoS One.* 2013;8(12):e85093. 10.1371/journal.pone.0085093 24386451 PMC3874034

[67] Adegbola AA Cox GF Bradshaw EM: Monoallelic expression of the human *FOXP2* speech gene. *Proc Natl Acad Sci U S A.* 2015;112(22):6848–54. 10.1073/pnas.1411270111 25422445 PMC4460484

[68] Ben-David E Shohat S Shifman S: Allelic expression analysis in the brain suggests a role for heterogeneous insults affecting epigenetic processes in autism spectrum disorders. *Hum Mol Genet.* 2014;23:4111–24. 10.1093/hmg/ddu128 24659497

[69] Ohlsson R Paldi A Graves JA: Did genomic imprinting and X chromosome inactivation arise from stochastic expression? *Trends Genet.* 2001;17(3):136–41. 10.1016/S0168-9525(00)02211-3 11226606

[70] Mo A Mukamel EA Davis FP: Epigenomic Signatures of Neuronal Diversity in the Mammalian Brain. *Neuron.* 2015;86(6):1369–84. 10.1016/j.neuron.2015.05.018 26087164 PMC4499463

[71] Lister R Mukamel EA Nery JR: Global epigenomic reconfiguration during mammalian brain development. *Science.* 2013;341(6146):1237905. 10.1126/science.1237905 23828890 PMC3785061

[72] Jaffe AE Gao Y Deep-Soboslay A: Mapping DNA methylation across development, genotype and schizophrenia in the human frontal cortex. *Nat Neurosci.* 2016;19(1):40–7. 10.1038/nn.4181 26619358 PMC4783176

[73] Szulwach KE Li X Li Y: 5-hmC-mediated epigenetic dynamics during postnatal neurodevelopment and aging. *Nat Neurosci.* 2011;14(12):1607–16. 10.1038/nn.2959 22037496 PMC3292193

[74] Kurukuti S Tiwari VK Tavoosidana G: CTCF binding at the H19 imprinting control region mediates maternally inherited higher-order chromatin conformation to restrict enhancer access to Igf2. *Proc Natl Acad Sci U S A.* 2006;103(28):10684–9. 10.1073/pnas.0600326103 16815976 PMC1484419

[75] Leighton PA Saam JR Ingram RS: An enhancer deletion affects both H19 and Igf2 expression. *Genes Dev.* 1995;9(17):2079–89. 10.1101/gad.9.17.2079 7544754

[76] Markenscoff-Papadimitriou E Allen WE Colquitt BM: Enhancer interaction networks as a means for singular olfactory receptor expression. *Cell.* 2014;159(3):543–57. 10.1016/j.cell.2014.09.033 25417106 PMC4243057

[77] Sanna CR Li WH Zhang L: Overlapping genes in the human and mouse genomes. *BMC Genomics.* 2008;9:169. 10.1186/1471-2164-9-169 18410680 PMC2335118

[78] McCole RB Oakey RJ: Unwitting hosts fall victim to imprinting. *Epigenetics.* 2008;3(5):258–60. 10.4161/epi.3.5.7052 18948747 PMC2814294

[79] Heintzman ND Stuart RK Hon G: Distinct and predictive chromatin signatures of transcriptional promoters and enhancers in the human genome. *Nat Genet.* 2007;39(3):311–8. 10.1038/ng1966 17277777

[80] Birnbaum RY Clowney EJ Agamy O: Coding exons function as tissue-specific enhancers of nearby genes. *Genome Res.* 2012;22(6):1059–68. 10.1101/gr.133546.111 22442009 PMC3371700

[81] Harmston N Lenhard B: Chromatin and epigenetic features of long-range gene regulation. *Nucleic Acids Res.* 2013;41(15):7185–99. 10.1093/nar/gkt499 23766291 PMC3753629

[82] Xu J Carter AC Gendrel AV: Landscape of monoallelic DNA accessibility in mouse embryonic stem cells and neural progenitor cells. *Nat Genet.* 2017;49(3):377–86. 10.1038/ng.3769 28112738 PMC5357084

[83] Cavalli G Misteli T: Functional implications of genome topology. *Nat Struct Mol Biol.* 2013;20(3):290–9. 10.1038/nsmb.2474 23463314 PMC6320674

[84] Misteli T: Beyond the sequence: cellular organization of genome function. *Cell.* 2007;128(4):787–800. 10.1016/j.cell.2007.01.028 17320514

[85] Parada LA McQueen PG Misteli T: Tissue-specific spatial organization of genomes. *Genome Biol.* 2004;5(7):R44. 10.1186/gb-2004-5-7-r44 15239829 PMC463291

[86] Foster HA Bridger JM: The genome and the nucleus: a marriage made by evolution. Genome organisation and nuclear architecture. *Chromosoma.* 2005;114(4):212–29. 10.1007/s00412-005-0016-6 16133352

[87] Meaburn KJ Agunloye O Devine M: Tissue-of-origin-specific gene repositioning in breast and prostate cancer. *Histochem Cell Biol.* 2016;145(4):433–46. 10.1007/s00418-015-1401-8 26791532 PMC4795970

[88] Meaburn KJ Gudla PR Khan S: Disease-specific gene repositioning in breast cancer. *J Cell Biol.* 2009;187(6):801–12. 10.1083/jcb.200909127 19995938 PMC2806312

[89] Stratigi K Kapsetaki M Aivaliotis M: Spatial proximity of homologous alleles and long noncoding RNAs regulate a switch in allelic gene expression. *Proc Natl Acad Sci U S A.* 2015;112(13):E1577–86. 10.1073/pnas.1502182112 25770217 PMC4386343

